# Supplementary material for: Better data for better predictions: data curation improves deep learning for sgRNA/Cas9 prediction
Source: PeerJ. 2026 Feb 10;14:e20706. doi: 10.7717/peerj.20706 (PMC12903899; doi:10.7717/peerj.20706)
Supplement: Supplemental Information 1 [file peerj-14-20706-s001.pdf]

SUPPLEMENTARY FIGURES

S1 Sequence similarity between each training set and held-out test set, as measured by Hamming distance

S2 Cross validation performance for crisprHAL$_{\text{Tev}}$, crisprHAL$_{\text{eSp}}$, and crisprHAL$_{\text{WT}}$ across tested training epochs

S3 eSpCas9 and SpCas9 $\log_2$FC score distributions for original and curated dataset versions

S4 Final control read count cutoff value TevSpCas9, eSpCas9, and SpCas9 $\log_2$FC score distributions versus a cutoff of 1

S5 Upstream and downstream di-nucleotide mean score plots for the *C. rodentium* TevSpCas9 dataset

S6 Upstream and downstream di-nucleotide mean score plots for the *E. coli* eSpCas9 dataset

S7 Upstream and downstream di-nucleotide mean score plots for the *E. coli* wild-type SpCas9 dataset

S8 crisprHAL$_{\text{eSp}}$ model performance on the hold-out and independent test sets using different input sequence lengths

S9 Pearson correlation metrics for crisprHAL$_{\text{Tev}}$, crisprHAL$_{\text{WT}}$, and prior models on the 3 independent test sets

S10 Benchmarking of the input sequence length and control condition read count cutoff using a prior 5-layer CNN architecture

SUPPLEMENTARY TABLES

S1 Final model architecture and parameters for crisprHAL$_{\text{Tev}}$, crisprHAL$_{\text{eSp}}$, and crisprHAL$_{\text{WT}}$

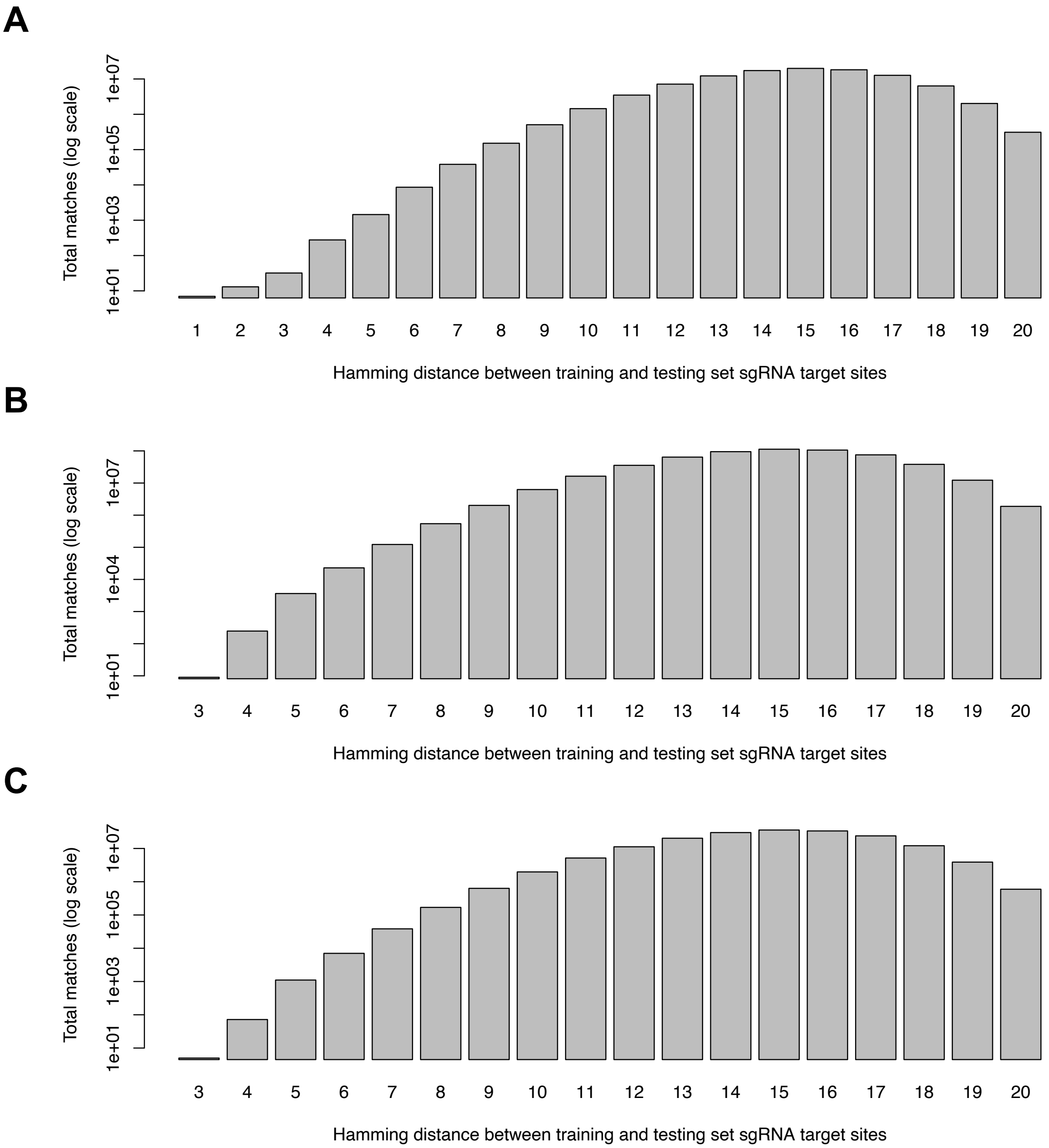

Figure S1. Summation of the training and held-out test set Hamming distances for the (A) *C. rodentium* TevSpCas9, (B) *E. coli* eSpCas9, and (C) *E. coli* wild-type SpCas9 datasets. Counts show the Hamming distance between each training set and each held-out testing set sgRNA target site. For the *E. coli* eSpCas9 and *E. coli* wild-type SpCas9 datasets, the maximum sequence similarity between any training and testing set sgRNA target site sequence was 3, with a maximum of 1 for the *C. rodentium* TevSpCas9 data. Total matches for each distance is shown on a $\log_{10}$ scale.

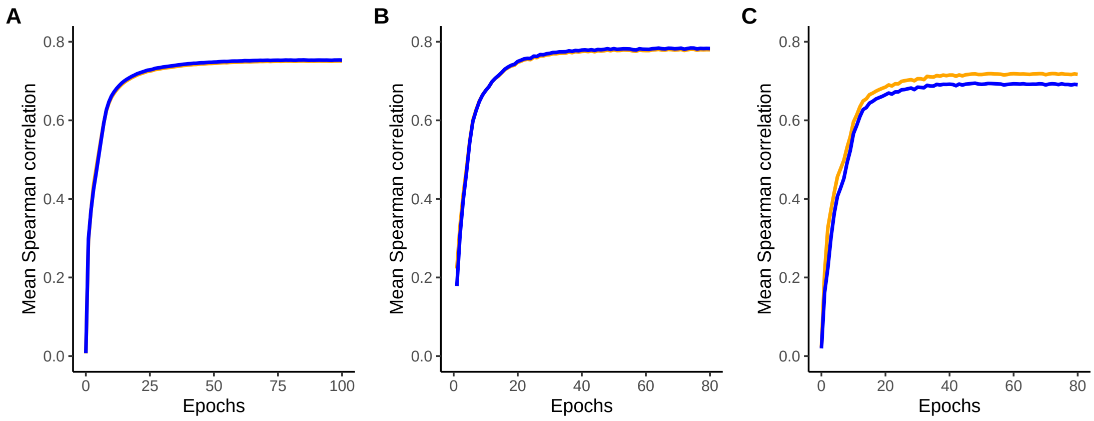

Figure S2. Mean 5-fold cross validation Spearman correlations (blue) and Pearson correlations (orange) for (A) crisprHAL$_{\mathrm{Tev}}$, (B) crisprHAL$_{\mathrm{eSp}}$, and (C) crisprHAL$_{\mathrm{WT}}$ across tested training epochs. The epoch providing the best mean Spearman correlation for each respective model is used for final model training.

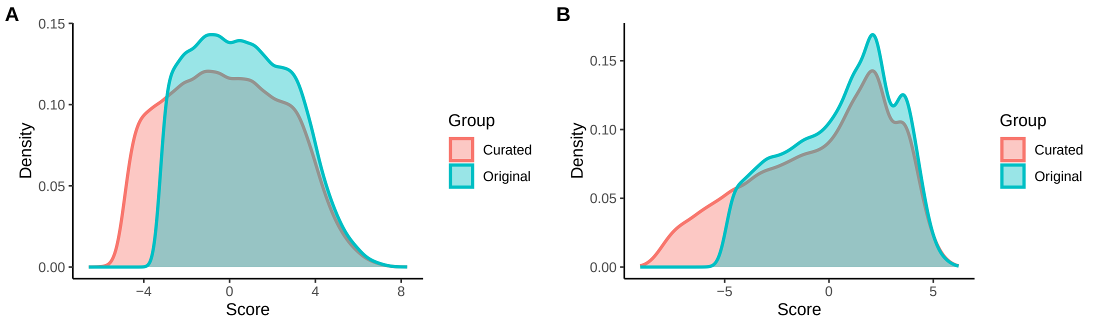

Figure S3. Densities of $\log_2$FC scores for the (A) *E. coli* eSpCas9 dataset and (B) *E. coli* wild-type SpCas9 dataset. The densities in orange show the distribution of the scores for each dataset following our data curation, with densities in blue showing the distribution of scores for data points in the original, prior, version of each dataset.

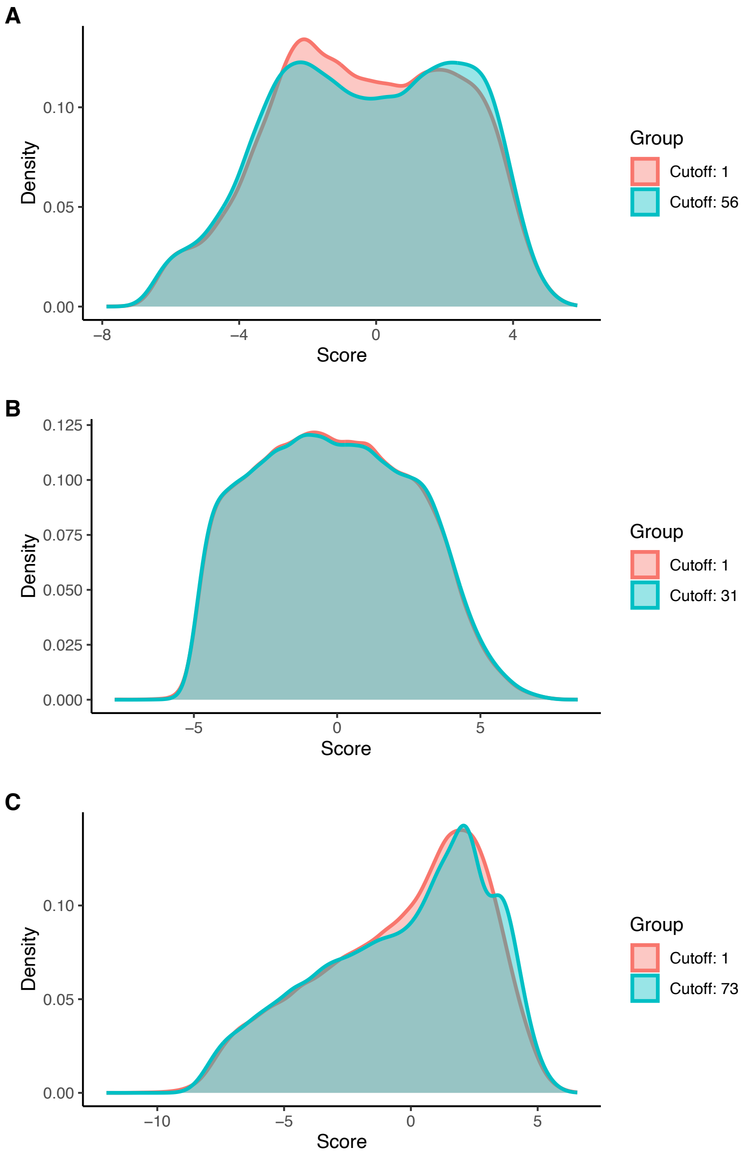

Figure S4. Densities of $\log_2$FC scores for the (A) *C. rodentium* TevSpCas9, (B) *E. coli* eSpCas9, and (C) *E. coli* wild-type SpCas9 datasets. The densities in blue show the distribution of $\log_2$FC scores for each dataset following our data curation using the dataset-specific control condition minimum read count cutoff, with densities in orange showing the distribution of $\log_2$FC scores using a cutoff value of 1.

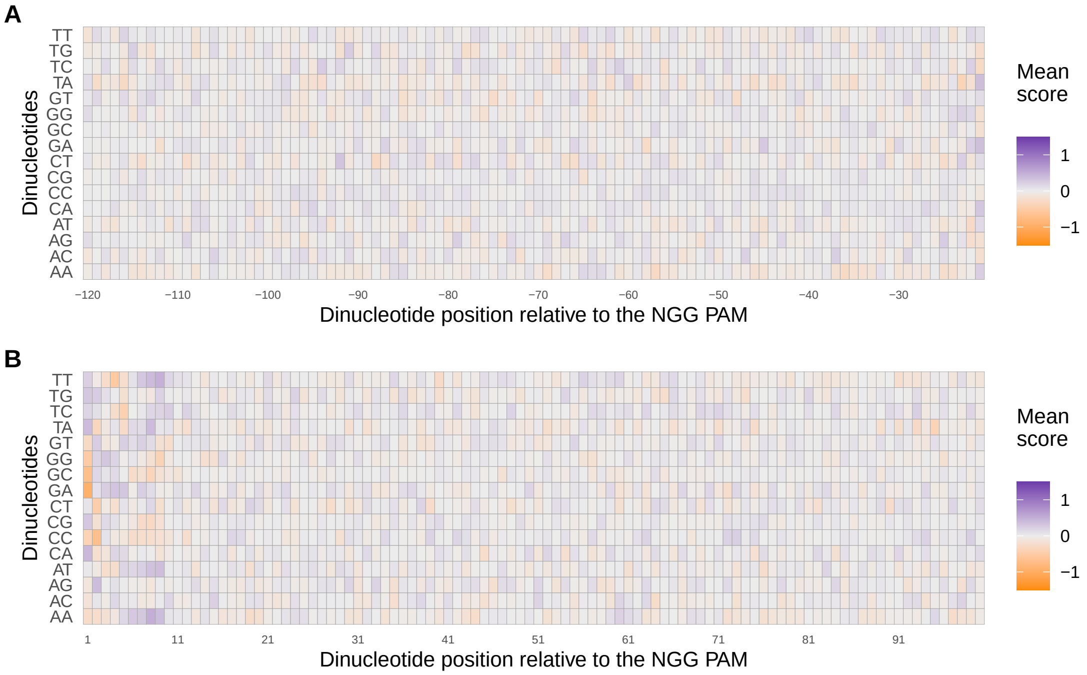

Figure S5. (A) Upstream and (B) downstream mean $\log_2$FC scores from the *C. rodentium* TevSpCas9 dataset for each di-nucleotide option adjacent to the target site and NGG PAM. Positions are labelled with respect to moving upstream or downstream of the PAM.

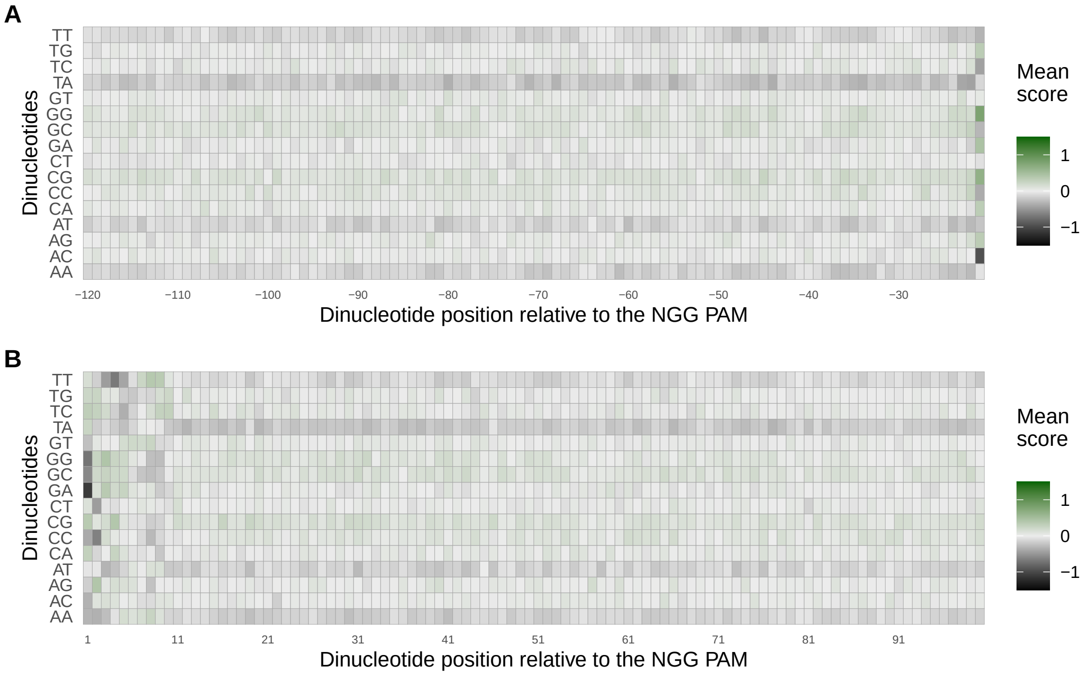

Figure S6. (A) Upstream and (B) downstream mean log_2FC scores from the *E. coli* eSpCas9 dataset for each di-nucleotide option adjacent to the target site and NGG PAM. Positions are labelled with respect to moving upstream or downstream of the PAM. Of note is the consistent disfavouring of AT-rich target site adjacent regions by eSpCas9.

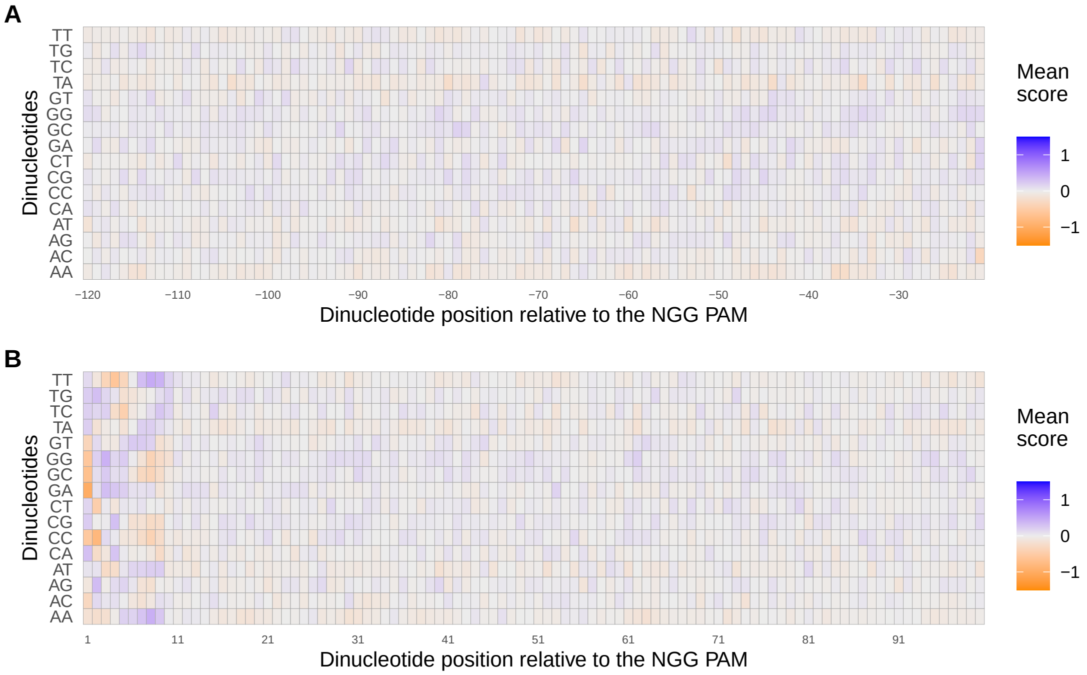

Figure S7. (A) Upstream and (B) downstream mean $\log_2$FC scores from the *E. coli* wild-type SpCas9 dataset for each di-nucleotide option adjacent to the target site and NGG PAM. Positions are labelled with respect to moving upstream or downstream of the PAM.

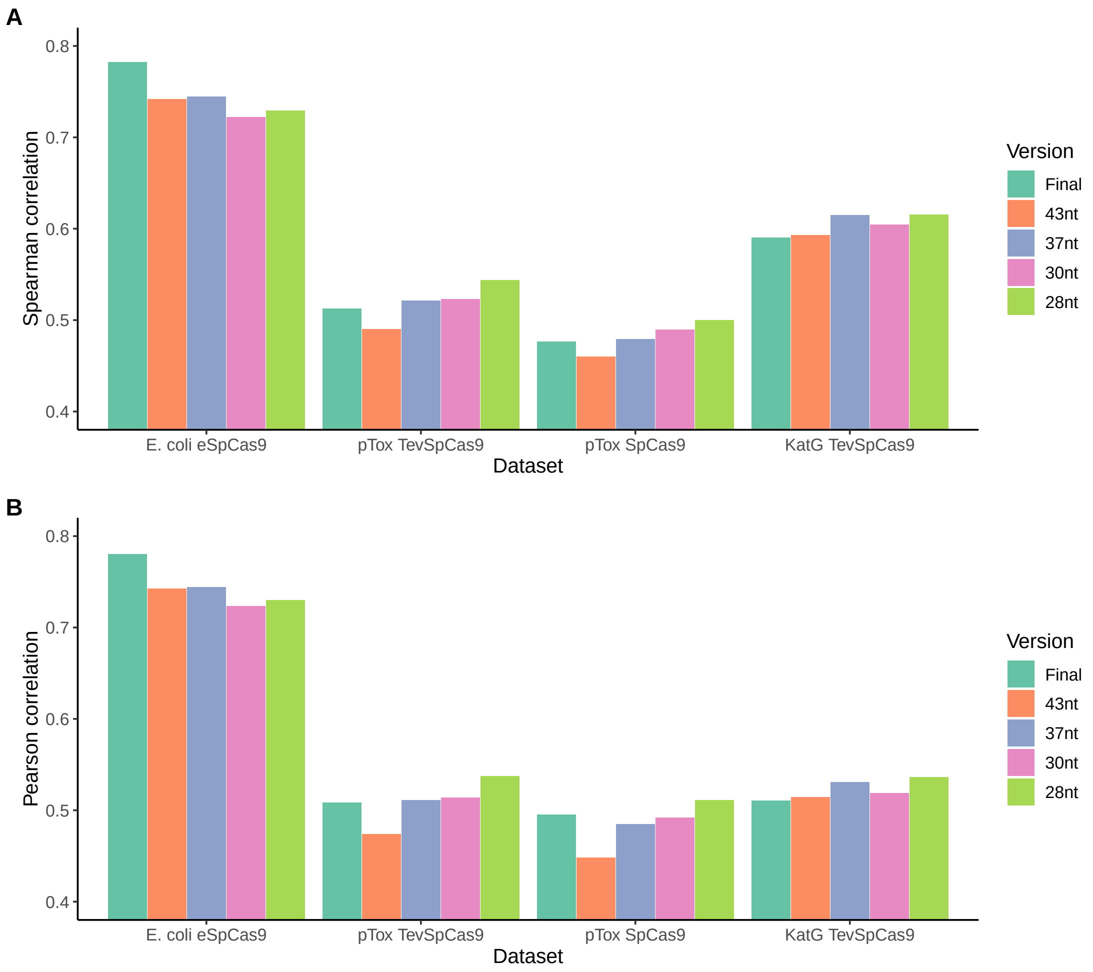

Figure S8. Performance from crisprHAL$_{eSp}$ model on the hold-out and independent test sets when using 5 different input sequence lengths, as measured by (A) Spearman correlation, and (B) Pearson correlation. The inputs tested with adjacent nucleotide inclusion (U=upstream, D=downstream) are: the final crisprHAL$_{eSp}$ 406 nt input (U=193, D=193), 43 nt used for DeepSgRNA$_{eSp}$ (U=10, D=10), 37 nt used for crisprHAL$_{Tev}$ (U=3, D=14), 30nt used for Guo$_{eSp}$ (U=4, D=6), and 28 nt used for crisprHAL$_{TL\text{-}Tev}$ and crisprHAL$_{TL\text{-}WT}$ (U=0, D=8). Given the unique target site adjacent nucleotide preferences by eSpCas9, the inclusion of these regions in the model input improves eSpCas9 performance, but hinders performance on wild-type SpCas9 and TevSpCas9 tasks. When the upstream nucleotides, and most downstream nucleotides, are excluded from the input, as per the 28 nt input sequence length, performance on the wild-type SpCas9 and TevSpCas9 test sets is improved.

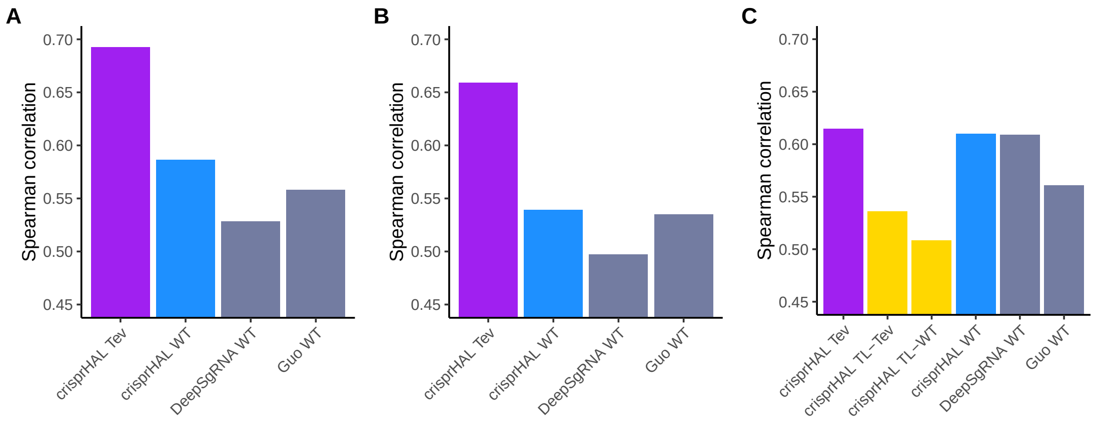

Figure S9. Pearson correlation model performance comparisons of crisprHAL$_{Tev}$ (purple) and crisprHAL$_{WT}$ (blue), the original crisprHAL$_{TL\text{-}Tev}$ and crisprHAL$_{TL\text{-}WT}$ models (gold), and the prior models, DeepSgRNA$_{WT}$ and Guo$_{WT}$ (grey) on (A) the *E. coli* pTox plasmid TevSpCas9 activity set, (B) the *E. coli* pTox plasmid SpCas9 activity set, and (C) the *S. enterica* KatG target in *E. coli* TevSpCas9 activity set.

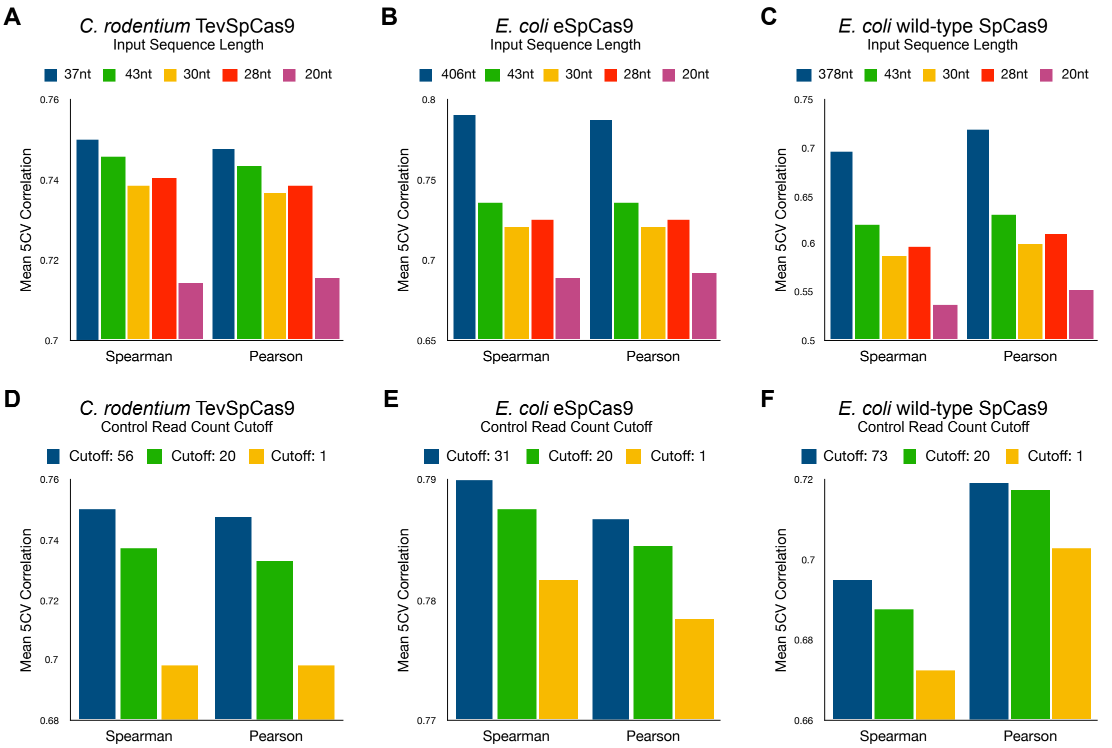

Figure S10. Mean 5-fold cross validation Spearman and Pearson correlation metrics using the 5-layer CNN architecture from the DeepSgRNA models, in place of the crisprHAL architecture, across the three training sets. (A-C) 5-layer CNN model performance across the following input sequence length tests: i) each dataset-specific input length (blue), ii) 43nt used by DeepSgRNA (green), iii) 30nt used by Guo (yellow), iv) 28nt used by the original crisprHAL (red), and the 20nt sgRNA target site as a baseline (purple). All input sequence length testing is performed with each dataset's respective control condition read count cutoff. (D-F) 5-layer CNN model performance using different control condition minimum read count cutoffs: each dataset-specific cutoff (blue), the previously used cutoff of 20 (green), and a baseline cutoff of 1 (yellow). All control read count cutoff testing is performed using the dataset-specific input sequence length.

Table S1. Architecture and parameters for the final models. Deep learning architecture, connections, and parameters for the crisprHAL$_{\text{Tev}}$, crisprHAL$_{\text{eSp}}$, and crisprHAL$_{\text{WT}}$ final models, using each model's respective input sequence length.

			crisprHAL Tev 37nt input		crisprHAL eSp 406nt input		crisprHAL WT 378nt input	
Layer type	Layer Name	Connected to	Output Shape	Param #	Output Shape	Param #	Output Shape	Param #
InputLayer	Input	-	(None, 37, 4)	0	(None, 406, 4)	0	(None, 378, 4)	0
Conv1D	c1	Input	(None, 37, 128)	1664	(None, 406, 128)	1664	(None, 378, 128)	1664
LeakyReLU	l1	c1	(None, 37, 128)	0	(None, 406, 128)	0	(None, 378, 128)	0
MaxPooling1D	p1	l1	(None, 19, 128)	0	(None, 203, 128)	0	(None, 189, 128)	0
Dropout	dr1	p1	(None, 19, 128)	0	(None, 203, 128)	0	(None, 189, 128)	0
Conv1D	c2	dr1	(None, 19, 128)	49280	(None, 203, 128)	49280	(None, 189, 128)	49280
LeakyReLU	l2	c2	(None, 19, 128)	0	(None, 203, 128)	0	(None, 189, 128)	0
MaxPooling1D	p2	l2	(None, 10, 128)	0	(None, 102, 128)	0	(None, 95, 128)	0
Dropout	dr2	p2	(None, 10, 128)	0	(None, 102, 128)	0	(None, 95, 128)	0
Conv1D	c3	dr2	(None, 10, 128)	49280	(None, 102, 128)	49280	(None, 95, 128)	49280
LeakyReLU	l3	c3	(None, 10, 128)	0	(None, 102, 128)	0	(None, 95, 128)	0
MaxPooling1D	p3	l3	(None, 5, 128)	0	(None, 51, 128)	0	(None, 48, 128)	0
Dropout	dr3	p3	(None, 5, 128)	0	(None, 51, 128)	0	(None, 48, 128)	0
Conv1D	c4	dr3	(None, 5, 128)	49280	(None, 51, 128)	49280	(None, 48, 128)	49280
LeakyReLU	l4	c4	(None, 5, 128)	0	(None, 51, 128)	0	(None, 48, 128)	0
MaxPooling1D	p4	l4	(None, 3, 128)	0	(None, 26, 128)	0	(None, 24, 128)	0
Bidirectional_LSTM	r1	dr1	(None, 256)	198144	(None, 256)	198144	(None, 256)	198144
Dropout	dr4	p4	(None, 3, 128)	0	(None, 26, 128)	0	(None, 24, 128)	0
Dropout	rd1	r1	(None, 256)	0	(None, 256)	0	(None, 256)	0
Flatten	f	dr4	(None, 384)	0	(None, 3328)	0	(None, 3072)	0
Flatten	f_LSTM	rd1	(None, 256)	0	(None, 256)	0	(None, 256)	0
Dense	d1	f	(None, 128)	49280	(None, 128)	426112	(None, 128)	393344
Dense	d1_LSTM	f_LSTM	(None, 128)	32896	(None, 128)	32896	(None, 128)	32896
LeakyReLU	ld1	d1	(None, 128)	0	(None, 128)	0	(None, 128)	0
LeakyReLU	ld1_LSTM	d1_LSTM	(None, 128)	0	(None, 128)	0	(None, 128)	0
Dropout	drd1	ld1	(None, 128)	0	(None, 128)	0	(None, 128)	0
Dropout	drd1_LSTM	ld1_LSTM	(None, 128)	0	(None, 128)	0	(None, 128)	0
Dense	d2	drd1	(None, 64)	8256	(None, 64)	8256	(None, 64)	8256
Dense	d2_LSTM	drd1_LSTM	(None, 64)	8256	(None, 64)	8256	(None, 64)	8256
LeakyReLU	ld2	d2	(None, 64)	0	(None, 64)	0	(None, 64)	0
LeakyReLU	ld2_LSTM	d2_LSTM	(None, 64)	0	(None, 64)	0	(None, 64)	0
Dropout	drd2	ld2	(None, 64)	0	(None, 64)	0	(None, 64)	0
Dropout	drd2_LSTM	ld2_LSTM	(None, 64)	0	(None, 64)	0	(None, 64)	0
Dense	x_1d_o	drd2	(None, 1)	65	(None, 1)	65	(None, 1)	65
Dense	x_LSTM_o	drd2_LSTM	(None, 1)	65	(None, 1)	65	(None, 1)	65
Concatenate	o_c	x_1d_o, x_LSTM_o	(None, 2)	0	(None, 2)	0	(None, 2)	0
Dense	o	o_c	(None, 1)	3	(None, 1)	3	(None, 1)	3
Total params				446469		823301		790533
Trainable params				446469		823301		790533
Non-trainable params				0		0		0
